# Correction: Endurance-trained subjects and sedentary controls increase ventricular contractility and efficiency during exercise: Feasibility of hemodynamics assessed by non-invasive pressure-volume loops

**DOI:** 10.1371/journal.pone.0321568

**Published:** 2025-04-01

**Authors:** Björn Östenson, Ellen Ostenfeld, Jonathan Edlund, Einar Heiberg, Håkan Arheden, Katarina Steding-Ehrenborg

In the Pressure-volume loop parameters subsection of the Materials and methods, there is an error in the first equation. The fraction of LVESP is incorrect. It should read 0.83 and 0.15. Please view the complete, correct equation here:


LVESP = 0.83 SBP + 0.15 DBP

There is an error in reference 18. The correct reference is: Westerhof N, Stergiopulos N, Noble MI, et al. Snapshots of Hemodynamics. Springer. 2010;2ndEd: 191.
