# Supplementary material for: Endurance-trained subjects and sedentary controls increase ventricular contractility and efficiency during exercise: Feasibility of hemodynamics assessed by non-invasive pressure-volume loops
Source: PLoS One. 2023 May 10;18(5):e0285592. doi: 10.1371/journal.pone.0285592 (PMC10171617; doi:10.1371/journal.pone.0285592)
Supplement: S1 Table — ET: endurance-trained subject; SC: sedentary control. (DOCX) [file pone.0285592.s001.docx]

| ^Slice number^  _Subject_ | 1 | 2 | 3 | 4 | 5 | 6 | 7 | 8 | 9 | 10 | 11 | 12 | 13 | 14 |
| --- | --- | --- | --- | --- | --- | --- | --- | --- | --- | --- | --- | --- | --- | --- |
| ET 1 | 102 | 116 | 108 | 97 | 120 | 116 | 103 | 118 | 110 | 98 | 119 | 90 | 102 |  |
| ET 2 | 101 | 98 | 89 | 102 | 94 | 101 | 98 | 100 | 94 | 100 | 84 | 102 |  |  |
| ET 3 | 68 | 72 | 66 | 68 | 67 | 72 | 71 | 73 | 70 | 73 | 72 | 74 |  |  |
| ET 4 | 86 | 83 | 85 | 87 | 86 | 88 | 89 | 88 | 103 | 87 | 86 | 90 | 87 |  |
| ET 5 | 88 | 84 | 91 | 85 | 91 | 86 | 93 | 86 | 85 | 86 | 87 | 83 | 89 |  |
| ET 6 | 84 | 87 | 72 | 82 | 70 | 88 | 83 | 81 | 73 | 79 | 75 | 85 |  |  |
| ET 7 | 85 | 90 | 76 | 83 | 77 | 86 | 81 | 85 | 79 | 85 | 79 | 83 |  |  |
| ET 8 | 96 | 95 | 81 | 99 | 94 | 70 | 111 | 97 | 78 | 103 | 96 | 71 | 101 | 97 |
| ET 9 | 100 | 95 | 99 | 95 | 89 | 87 | 103 | 97 | 96 | 97 | 95 | 95 | 94 |  |
| ET 10 | 106 | 100 | 96 | 83 | 101 | 94 | 85 | 92 | 89 | 94 |  |  |  |  |
| ET 11 | 101 | 96 | 80 | 81 | 76 | 62 | 94 | 89 | 77 | 96 | 88 | 95 |  |  |
| ET 12 | 79 | 81 | 85 | 80 | 80 | 82 | 78 | 74 | 85 | 84 | 80 | 85 | 81 |  |
| ET 13 | 71 | 62 | 64 | 61 | 74 | 61 | 70 | 73 | 74 | 65 | 69 | 66 | 70 |  |
| SC 1 | 81 | 86 | 89 | 94 | 90 | 89 | 82 | 89 | 80 | 84 | 85 | 91 | 86 | 91 |
| SC 2 | 131 | 102 | 84 | 127 | 123 | 120 | 115 | 136 | 130 | 106 | 138 | 134 |  |  |
| SC 3 | 85 | 66 | 83 | 68 | 93 | 78 | 75 | 64 | 81 | 67 | 87 | 71 | 86 |  |
| SC 4 | 101 | 83 | 104 | 78 | 112 | 74 | 101 | 73 | 115 | 76 | 107 | 74 |  |  |
| SC 5 | 78 | 85 | 81 | 85 | 82 | 89 | 84 | 86 | 83 | 88 | 87 | 88 |  |  |
| SC 6 | 58 | 68 | 66 | 72 | 66 | 69 | 62 | 72 | 64 | 65 | 62 | 68 | 62 |  |
| SC 7 | 92 | 94 | 95 | 95 | 107 | 106 | 100 | 100 | 102 | 98 | 103 | 103 |  |  |
| SC 8 | 98 | 82 | 81 | 79 | 81 | 82 | 102 | 77 | 106 | 90 | 101 | 86 | 71 | 84 |
| SC 9 | 105 | 97 | 102 | 94 | 105 | 92 | 93 | 85 | 86 | 81 | 92 | 85 | 97 |  |
| SC 10 | 79 | 93 | 89 | 79 | 97 | 96 | 89 | 94 | 94 | 88 | 92 | 93 | 94 | 94 |
