# Supplementary material for: Endurance-trained subjects and sedentary controls increase ventricular contractility and efficiency during exercise: Feasibility of hemodynamics assessed by non-invasive pressure-volume loops
Source: PLoS One. 2023 May 10;18(5):e0285592. doi: 10.1371/journal.pone.0285592 (PMC10171617; doi:10.1371/journal.pone.0285592)
Supplement: S2 Table — ET: endurance-trained subject; SC: sedentary control. (DOCX) [file pone.0285592.s002.docx]

| ^Exercise bout^  _Subject_ | 1 | 2 | 3 | 4 | 5 | 6 | 7 | 8 | 9 | 10 |
| --- | --- | --- | --- | --- | --- | --- | --- | --- | --- | --- |
| ET 1 | 118/74 | 109/65 | 124/78 | 114/67 | 115/81 |  |  |  |  |  |
| ET 2 | 126/69 | 131/77 | 124/82 | 128/79 | 120/76 | 118/74 | 118/75 | 126/72 |  |  |
| ET 3 | 114/73 | 116/75 | 120/68 | 116/64 | 116/64 | 100/58 | 115/58 | 106/62 |  |  |
| ET 4 | 130/77 | 128/79 | 138/72 | 136/82 | 134/81 | 131/82 | 131/75 | 127/84 |  |  |
| ET 5 | 120/66 | 117/67 | 117/73 | 122/69 | 118/71 | 120/72 | 128/60 | 116/65 |  |  |
| ET 6 | 153/117 | 155/76 | 149/104 | 163/100 | 175/111 | 154/93 | 168/105 | 150/93 |  |  |
| ET 7 | 142/88 | 131/97 | 137/92 | 149/91 | 137/93 | 146/93 | 136/84 | 142/89 |  |  |
| ET 8 | 127/93 | 131/93 | 136/104 | 141/98 | 134/84 | 134/78 |  |  |  |  |
| ET 9 | 103/63 | 100/63 | 108/57 | 105/61 | 110/60 | 103/59 | 104/56 | 103/61 |  |  |
| ET 10 | 127/56 | 123/58 | 133/51 | 113/59 | 124/50 | 110/52 | 124/44 | 112/63 | 116/45 |  |
| ET 11 | 158/97 | 173/99 | 165/91 | 162/92 | 167/97 |  |  |  |  |  |
| ET 12 | 130/61 | 140/89 | 137/88 | 137/92 | 138/70 |  |  |  |  |  |
| ET 13 | 125/65 | 118/66 | 114/76 | 122/66 | 124/66 | 121/61 | 125/65 | 120/63 |  |  |
| SC 1 | 116/75 | 129/67 | 141/88 | 131/69 | 124/64 | 120/66 | 120/73 | 126/71 | 120/63 |  |
| SC 2 | 136/76 | 131/59 | 122/80 | 129/81 |  |  |  |  |  |  |
| SC 3 | 124/65 | 126/89 | 125/96 | 109/79 | 125/75 | 118/49 | 112/80 | 120/65 | 117/81 | 119/84 |
| SC 4 | 112/74 | 129/90 | 148/78 | 141/83 | 140/78 | 111/64 | 113/65 |  |  |  |
| SC 5 | 134/81 | 146/77 | 149/83 | 146/88 | 147/84 | 140/71 | 126/89 |  |  |  |
| SC 6 | 135/70 | 144/62 | 135/72 | 140/69 | 138/83 | 144/68 | 137/64 |  |  |  |
| SC 7 | 98/51 | 101/50 | 93/54 | 113/44 | 118/50 | 104/51 | 104/45 | 104/49 |  |  |
| SC 8 | 152/81 | 143/84 | 142/90 | 130/84 | 123/81 | 141/81 | 133/96 | 147/86 |  |  |
| SC 9 | 127/75 | 124/77 | 129/74 | 130/77 | 123/62 | 123/59 | 127/60 | 115/62 |  |  |
| SC 10 | 166/104 | 156/108 | 149/97 | 145/106 | 146/101 | 150/97 |  |  |  |  |
