# Supplementary material for: Endurance-trained subjects and sedentary controls increase ventricular contractility and efficiency during exercise: Feasibility of hemodynamics assessed by non-invasive pressure-volume loops
Source: PLoS One. 2023 May 10;18(5):e0285592. doi: 10.1371/journal.pone.0285592 (PMC10171617; doi:10.1371/journal.pone.0285592)
Supplement: Attachment — Submitted filename: Review of CMR PVloops.docx [file pone.0285592.s003.docx]

Review of ”*Endurance-trained subjects and sedentary controls increase ventricular contractility and efficiency during exercise–feasibility of hemodynamics assessed by non-invasive pressure-volume loops”* by Östenson et al.

In the above-mentioned study, the authors assessed endurance-trained athletes and sedentary controls at rest and during exercise with non-invasive pressure-volume loops generated by CMR and NIBP using Segment (a software from Medviso, Sweden).

The aim of the study was to assess if pressure-volume loops could be obtained during exercise.

Twenty-three subjects, 13 endurance-trained athletes, and 10 sedentary controls matched for age and gender were included. They were examined at rest and at moderate exercise intensity.

Pressure volume loops at rest and during exercise were computed using input data from a combination of volumetric measurements obtained from the CMR data and ESP from non-invasive brachial blood pressure. End-diastolic pressure was inputted by the user.

End-systolic elastance (Ees) and Effective arterial elastance (Ea) were found using single-beat technique.

The authors concluded that the method is feasible and a new useful tool to assess hemodynamics even during exercise.

The manuscript is well written. The research group is well known for its CMR capabilities.

Cardiac function and physiology have been studied intensively for decades. Pressure-Volume loops are an accurate method to describe cardiac function. The gold standard for obtaining PV-loops is measuring pressure and volume simultaneously in the ventricle using an admittance/conductance catheter. Combining CMR (the gold standard for volume assessment) with pressure has great potential in research and in the clinic.

Therefore, the method proposed by this group of researchers is of great interest.

I do have some limitations.

1. The equations used in the methods section are difficult to follow.

In order to increase impact and readability may I suggest using End-systolic pressure (ESP) instead of *LVP max elastance.* To use end-systolic volume (ESV) instead of *LVV max elastance*.

The authors use EDV for end-diastolic volume in equation 6 already.

Like Borlaug and Kass in fig 1.

Figure 1 Heart Fail Clin 2009;5(2):217-28

2. Why don’t the authors rewrite equation 11 to EA/EES = ESV/SV, which is extremely precise using CMR. (and closely related to LVEF).
3. I must admit I have problems with the user input for EDP. It is a guess. LVEDP can be above 15 mmHg, especially in decompensated heart failure patients.
4. Brachial blood pressure was measured non-invasively. However, it is unclear if the pressure was
   a. derived using pulse-wave analysis, which reflects central blood pressure and hence ESP, or
   b. from standard brachial sphygmomanometry, which doesn’t reflect central BP/ESP and has for many decades been a poor surrogate for aortic pressure due to pressure amplification (fig 2 McEniery et al). If so, it is a limitation and should be addressed.
      i. In the case of the later multiplication of 0.9 has been used to derive ESP.

Figure 2 Pressure amplification Eur Heart J. 2014 Jul 7; 35(26): 1719–1725.

5. For equation 9 (the standard SVR equation), the authors assume CVP is = 0 mmHg. CVP is rarely 0 mmHg, and I think it is an error to do so. The equation should be rewritten to MAP/CO = total peripheral resistance.
6. The authors refer to reference 9 and 10, where they have demonstrated the agreement of PV-loops generated by invasively measured pressures and CMR volumetric data compared to PV-loops generated conductance catheters in pigs. Has the non-invasive method (used here) been compared to an invasive method in humans? Ea/Ees ratio is very precise in CMR due to the relationship between ESV and SV (see 2), but we don’t know about the individual components (Ea and Ees). The real goal is to compare multiple non-invasive generated PV-loops during changes in preload or afterload.
