# Supplementary material for: Endurance-trained subjects and sedentary controls increase ventricular contractility and efficiency during exercise: Feasibility of hemodynamics assessed by non-invasive pressure-volume loops
Source: PLoS One. 2023 May 10;18(5):e0285592. doi: 10.1371/journal.pone.0285592 (PMC10171617; doi:10.1371/journal.pone.0285592)
Supplement: Attachment — Submitted filename: Response to Reviewers.docx [file pone.0285592.s004.docx]

**Point-by-Point Response to Editor and Reviewers**

PONE-D-22-29573

Endurance-trained subjects and sedentary controls increase ventricular contractility and efficiency during exercise–feasibility of hemodynamics assessed by non-invasive pressure-volume loops

PLOS ONE

Dear Hans-Peter Kubis, PD. Dr. rer. nat.,

Thank you for carefully reviewing our submitted manuscript. We have read and addressed all concerns presented by the reviewers. Please find our point-by-point comments below with excerpts from the amended manuscript, where applicable.

Yours sincerely,

Björn Östenson, M.D., PhD student, First Author

Katarina Steding-Ehrenborg, Associate Professor, PhD, Last Author

**Reviewer 1:** The work conducted by the authors is novel and should be commended. This is a well written manuscript and demonstrates the feasibility of this novel methodology in both trained and untrained participants.

The methods and experimental protocol have been performed by an experienced group and the data presented clearly.

I have no major issues to raise within my review.

My minor points are as follows:

**R1C1:** Title: I would recommend the use of a colon before ‘feasibility of hemodynamics…’. This would be more appropriate here.

**Response:** Thank you for pointing out a grammatical improvement to the title. We have now updated the title according to the suggestion (page 1, lines 4 – 6, “Endurance-trained subjects and sedentary controls increase ventricular contractility and efficiency during exercise*:* feasibility of hemodynamics assessed by non-invasive pressure-volume loops”)

**R1C2:** Study population: Please note which sports or modality of endurance training your cohort were undertaking because there can be subtle differences in cardiac adaptations between sports. I.e. swimming vs. land-based exercise, or running vs. rowing.

**Response:** All athletes were triathletes. This is now clarified on page 5, line 75 – 77.

**Updated text:** Thirteen ET subjects *from a local triathlon association* with median age 48 years [interquartile range (IQR) 34–60, one female] and ten healthy sedentary controls (SC) matched for age and sex (age 43 years [27–57], one female) were included.

**R1C3:** [Cardiac magnetic resonance imaging and exercise protocol:] The use of breath-holds for cardiac image acquisition will briefly increase brachial blood pressures. Indeed, this potentially appears evident in Supplemental table 2 with some fairly large fluctuations in systolic blood pressure between exercise bouts.

It appears that blood pressure measures were taken simultaneously with cardiac image acquisition. Please clarify whether there was a specific order for breath holds and brachial blood pressure measures, whether they were separated, or collected was simultaneously. Additionally, please clarify (if you made note) whether there was consistency of this order between subjects.

**Response:** We have updated the Cardiac magnetic imaging and exercise protocol section under Materials and methods (page 7, lines 127 – 131)

**Updated text:** *For all participants, blood pressure measurement was initiated immediately after exercise cessation using an automatic brachial sphygmomanometer (Invivo Expression, Invivo Corporation, Orlando, FL, USA), followed by breath-hold and image acquisition during the blood pressure measurement.*

**R1C4 (part 1):** [Cardiac magnetic resonance imaging and exercise protocol:] What determined one or three slices being acquired, and when the participant began exercise again? I think this should be stated here. Was it a heart rate dropping below 55% of their predicted max, or something different?

**Response:** This has been clarified on (page 7, lines 117 – 127).

**Updated text:** Each breath-hold image acquisition was set to last 5 – 15 seconds *by modifying the number of concatenations of the sequence. Shorter breath-holds resulted in fewer acquired short-axis slices per exercise bout compared to longer breath-holds. The number of concatenations was individually modified to adjust for* individual breath-hold capacity of the study participants. Typically, one to three short-axis slices were acquired during cessation in the scanner before the next exercise session started. *A new exercise bout during incremental load was initiated immediately after image acquisition from a low starting load until target intensity was achieved again.* This *process* was repeated until images of the whole ventricle were acquired. Heart rate was noted for each image slice (S1 Table). Mean exercise heart rate was calculated by summation of heart rates for each imaging slice divided by the number of image slices.

**R1C4 (part 2):** Also, were all imaged acquired at a heart rate of 55% predicted maximum or above? Three hearts rates in Supplemental table 1 are below 60 bpm and many are 61-70 bpm. Three incorrect heart rates are also included in the S1 table, two for participant ET 1 and one for ET 7.

**Response:** The target of 60% MPHR was achieved during exercise. However, the heart rate decreased upon exercise cessation when images were acquired (presented data), dropping below the target of 60% MPHR. The reviewer is correct in identifying the outlier heart rates. We have reanalyzed every exercise CMR short-axis slice used for volume quantification and updated Supplemental table 1 with the correct values. We have also updated the Limitations section to address the drop in heart rate during image acquisition (pages 25 – 26, lines 512 – 518).

**Updated text:** *The CMR imaging protocol used included cine balanced steady state free precession sequences, requiring exercise cessation and breath-holds during image acquisition. The consequence of the exercise cessation was a drop in heart rate during image acquisition and although the target heart rate was reached during exercise, image acquisition was actually performed at slightly lower heart rates. Thus, for future studies using exercise cessation the heart rate during exercise should be higher than target heart rate in order ensure image acquisition occurs at the target heart rate.*

**R1C5:** [Cardiac volumes at rest and exercise:] Inconsistent percentage increases during exercise between the text (line 217) and table 2.

**Response:** Thank you for pointing out the inconsistency between text in Results section and Table 2. We have checked the data and updated the text with correct values (page 13, lines 255 – 258).

**Updated text:** Cardiac volumes at rest and during exercise are presented in Table 2. Heart rate increased as expected in ET (*67%* increase) and in SC (*41%* increase). Systolic blood pressure increased by 10% in ET and by 14% in SC. Also, rate pressure product increased by *92%* in ET and by *74%* in SC.

**R1C6:** [Discussion:] The first part of the sentence starting on line 315 needs to be more specific in order to make the conclusion in the latter part, rather than ‘…in line with widely known and accepted exercise physiology…’. Looking at the references, 3 and 5 appear to contract one another in their findings of SV augmentation via EDV increase or ESV reduction. Given this this section is opening your key findings, I feel that these supporting statements should be clearer as to what you are referring to.

**Response:** We have updated the sentence about previous physiological findings in the beginning of the Discussion section (page 19, lines 364 – 371) to be more specific.

**Updated text:** The *physiological exercise response of LV volumes is* in line with *previous studies, demonstrating increased LVSV through increased LVEDV and decreased LVESV in ET, and increased LVSV through maintained or decreased LVEDV and decreased LVESV in healthy volunteers [3,5,6]. Furthermore, the physiological exercise response of hemodynamics is also in line with previous studies, demonstrating decreased SVR and increased contractility [30,31],* indicating that non-invasively obtained PV loops during exercise can be used to assess hemodynamic changes.

**R1C7:** [Limitations:] Please add the influence of breath holds on blood pressure as a study limitation as per point 3 of this review.

**Response:** We have added a paragraph about the influence of breath holds on blood pressure to the Limitations section (page 26, lines 518 – 521).

**Updated text:** *The breath-holds may influence the systolic and diastolic blood pressure, as they have been found to increase with prolonged apnea [52,53].* *However, the breath-holds were typically 8 – 12 seconds and any apnea-induced increase in blood pressure would therefore be expected to be minor.*

Reviewer 2: In this manuscript, Ostenson and colleagues sought to determine whether pressure-volume (PV) analysis of resting and exertional ventricular function can be derived noninvasively from a combination of cardiac MR (CMR) and brachial blood pressure. Thirteen endurance trained athletes and ten controls underwent noninvasive hemodynamic assessment during rest and moderate intensity during supine exercise at 60% maximum predicted heart rate (MPHR). Authors nicely demonstrate the feasibility/success of noninvasively deriving PV loops to facilitate enhanced understanding of ventricular function. Overall, I read this manuscript with enthusiasm. This manuscript has enormous potential; while not yet ready for publication in its current form, I ask the authors to consider comments below as constructive criticisms to further develop what could be a very nice manuscript that would sit very well in the literature, the limitations of this model notwithstanding.

**R2C1:** In study population, include how many males/females were included in the ET group, since authors indicate the control population was age/sex matched and included gender data for the controls. These data are included neither in the body of the text nor in table 1.

**Response:** There was one female endurance-trained subject and one female control in the study population. This is reported under Study population, (page 4, lines 77 – 78) and now also clarified in Table 1.

**Updated table:**

**Table 1. Subject characteristics.**

|  | ET (n=13) | SC (n=10) |
| --- | --- | --- |
| *Female (n, %)* | *1 (8 %)* | *1 (10%)* |
| Age (years) | 48 [34-60] | 43 [27-57] |
| Height (cm) | 180 [178-186] | 180 [175-184] |
| Weight (kg) | 77 [69-83] | 81 [75-85] |
| BMI (kg/m^2^) | 23.5 [21.3-25.4] | 24.9 [23.5-25.4] |
| BSA (m^2^) | 1.96 [1.85-2.08] | 1.99 [1.89-2.09] |
| Training Volume (h/week) | 6 [4.4-7.5] * | 1 [0.4-2] |

Number of females with percentage in parenthesis; other variables expressed as median values [IQR].

BMI: body mass index; BSA: body surface area; ET: endurance-trained subjects; SC: sedentary controls.

* p<0.05 ET vs SC.

**R2C2:** What do authors mean by “sample size was determined by feasibility”? This is a highly confusing statement. I think the authors mean to indicate that this was an exploratory analysis. The sentence in its current form should be removed and authors should write something to the effect of, “In this pilot analysis, sample size was limited to demonstrate feasibility of non-invasive derivation of PV loops”. Or, remove the sentence entirely.

**Response:** We have updated the Materials and methods section with a clearer statement about how sample size was determined (page 5, lines 93 – 94).

**Updated text:** *Sample size was limited in this pilot analysis to demonstrate feasibility of non-invasive PV loops using CMR.*

**R2C3 (Part 1):** In abstract, authors indicate that exercise was completed at 60% MPHR, but in the methods section, authors indicate exercise was at 55-70% MPHR. Please clarify what was the exercise intensity for the sake of consistency.

**Response:** We have updated the Methods and materials section so that it consistently refer to the target heart rate as 60 % of maximum predicted heart rate (MPHR) (page 6, lines 109 – 111).

**Updated text:** Study participants bicycled during incremental load *from a low starting load* until achieving moderate exercise intensity, defined as *60%* of estimated maximum heart rate calculated as 220 beats per minute minus age [15].

**R2C3 (Part 2):** Along these lines, I am confused by the data presented and it’s not clear to me that the intended exercise intensity was achieved. The median age for ET group was 48. 220-48 = 172bpm, so the MPHR for ET group is 172bpm. The HR during exercise (table 2) is 77bpm for this group. 77bpm represents 44% of the MPHR. Authors indicate that exercise was completed at 60% MPHR in the abstract, or 55-70% MPHR in the methods section (see above statement on this discrepancy). A HR of 77bpm is nowhere near 60% MPHR. Please clarify this discrepancy. Similar for the SC group.

**Response:** The target of 60% MPHR was achieved during exercise. As pointed out, the median age for ET group was 48 years, corresponding to an MPHR of 172 bpm and 60% MPHR of 103 bpm. The ET group cycled until they reached a median target heart rate of 100 bpm (not presented data). However, the heart rate decreased upon exercise cessation when images were acquired (presented data), dropping below the target of 60% MPHR. We have updated the Limitations section to clarify (page 25 – 26, lines 512 – 518).

**Updated text:** *The CMR imaging protocol used included cine balanced steady state free precession sequences, requiring exercise cessation and breath-holds during image acquisition.* *The consequence of the exercise cessation was a drop in heart rate during image acquisition and although the target heart rate was reached during exercise, image acquisition was actually performed at slightly lower heart rates. Thus, for future studies using exercise cessation the heart rate during exercise should be higher than target heart rate in order ensure image acquisition occurs at the target heart rate.*

**R2C4:** Authors nicely and succinctly describe some of the benefits and limitations of noninvasive PV analysis. However, there additional limitations which need to be included. First, is the physiology of supine v. upright hemodynamic assessments. Exercise hemodynamics vary depending on the presence/absence of a gravitational load – ie, exercise in a supine v. upright position, as does work intensity of the skeletal muscle – e.g. reliance on muscle groups during supine exercise that are not engaged to the same intensity during upright exercise. As such, hemodynamic abnormalities occurring during upright exercise likely are not adequately redemonstrated by conducting a hemodynamic assessment in a supine position. This issue really needs to be discussed. The second consideration is cost, since cost associated with cardiac MR is not inexpensive. Along these lines is the time required to complete this type of test and the necessity of having MRI-compatible equipment available – ie, having an ergometer available that is MRI compatible.

**Response:** We have updated the Limitations section to include discussions on the influence of body position on hemodynamics, and the cost effectiveness and availability of the method proposed (page 26 – 27, lines 528 – 539).

**Updated text:** *The study participants exercised in the CMR scanner using a cycle ergometer in the supine position. Supine body position influence hemodynamics by means of changed blood distribution, leading to greater filling pressures compared to upright body position [55]. Supine exercise has less recruited muscle mass and leg perfusion pressure gradient compared to upright exercise, resulting in lower peak VO2, heart rate, and workload [56,57]. Thus, hemodynamic abnormalities found during upright exercise may not be adequately comparable with supine exercise results using the proposed method from this study.*

*A limitation to the method, although not a limitation of the study, is the requirement of a CMR scanner with an MRI compatible cycle ergometer. The equipment and adequate expertise are expensive and of limited availability. Additionally, the time required to conduct the test and analyze the data is not insignificant.*

**R2C5:** As a follow-up, do the authors envision that this technique be used for research purposes only, or for clinical purposes as well? I hope it is the latter, since there is enormous potential for this technique to advance the understanding of mechanisms of functional impairments among individuals with cardiovascular and pulmonary vascular disease – both impacting the LV and RV. For this reason, authors need to expand on the section “Considerations when using non-invasive pressure-volume loops from exercise CMR” to include the above-stated issues.

**Response:** We have further expanded on the section “Considerations when using non-invasive pressure-volume loops from exercise CMR” to include a discussion on the proposed method’s clinical utility (page 21, lines 406 – 410).

**Updated text:** *This method may be clinically useful for diagnosis and prognosis of different cardiovascular diseases that can be unmasked by exercise. Exercise CMR is currently used mainly for research purposes, but with technical advancements we expect this method to be increasingly available clinically in the near future.*

**R2C6:** Authors also need to discuss the feasibility of RV PV analysis, albeit briefly, since the focus of this paper is not on RV hemodynamics, but nonetheless, there have been a large number of manuscripts addressing RV PV analysis, and at least in the United States, both the American Heart Association, and the NIH/NHLBI have published scientific statements in the past several years emphasizing the need to further study and characterize RV physiology/pathophysiology. This manuscript has the potential to sit very well given the current landscape of understanding LV and RV physiology/pathophysiology.

**Response:** We have added a brief discussion on the feasibility of RV PV loop analysis (page 21, lines 414 – 418).

**Updated text:** *It also needs to be emphasized that the present study was limited to the LV hemodynamics only. There is a need to study right ventricular (RV) physiology in order to increase the understanding of RV pathology. The proposed method could, if tweaked and validated for the RV in a future study, potentially characterize biventricular hemodynamics.*

**R2C7:** The manuscript would benefit from highlighting the added value of PV analysis compared to traditional invasive hemodynamic analysis with a Swan-Ganz catheter. For example, looking at Tedford’s RV PV exercise data in PH patients (Circ HF 2013, Circ 2016), or Cornwell’s RV PV data in healthy individuals (J. Physiology 2020). Limited data are derived from showing that right atrial pressure increases during exercise among patients with PH and RV dysfunction; similarly, limited insight results from completing an invasive CPET on normal healthy individuals demonstrating that RAP is normal. However, RV PV data in healthy individuals demonstrate significant contractile reserve, which is not adequately demonstrated simply by looking at RAP, or even by a stress echo demonstrating “normal ventricular function”. These examples are meant to be illustrative considerations for the authors to include a discussion on this subject, further supporting their argument as to the potential relevance of the novel method they have described in this manuscript. The discussion section should include mention of these concepts.

**Response:** We have added a new Discussion subsection titled “Comparing non-invasive and invasive pressure-volume loops” to address the advantages with the proposed method compared to traditional invasive hemodynamic analysis (page 20, lines 382 – 396).

**Updated text:** The method of assessing PV loops non-invasively has some advantages compared to the invasive counterpart. Complications of cardiac catheterization include vascular and major incidences, for example hematoma, infection, embolization, and myocardial infarction [37]. In contrast, the method of deriving PV loops non-invasively from exercise CMR comes with the same low risks as an ordinary CMR scan. Although catheterization can provide direct measurements of chamber pressures, the added value of, for example, increased atrial pressures during exercise is limited [38]. While right atrial pressure is demonstrated normal in healthy individuals during excise, right atrial pressure increases during exercise among patients with pulmonary hypertension, and increased left atrial pressures should be excluded in the diagnosis of precapillary pulmonary hypertension [39]. In addition, the contractile reserve, which can be analyzed from both invasive and non-invasive LV PV loops, provide significant information about cardiac hemodynamics, which is not adequately demonstrated simply by looking at atrial pressures [40]. Thus, non-invasive PV loops from exercise CMR is a safe method providing relevant data about hemodynamics.

**R2C8:** The discussion section on afterload (EA) should be elaborated on. Authors should review J. Mitchell’s JACC 1994 paper (JACC 1994; 24: 845-849), demonstrating the differences in hemodynamic response to dynamic v. static exercise. It is not surprising that authors found in this manuscript that Ea was reduced during dynamic exercise.

**Response:** We have elaborated on and updated the discussion section on arterial elastance (E_A_) (page 23, lines 442 – 452).

**Updated text:** The effect on arterial elastance (E_A_) has differed between previous exercise studies, using both invasive and non-invasive measurements [30,31,44]. The results from the present study are in line with Asanoi et al. [30] who found *invasive* EA to decrease during aerobic and anaerobic *supine cycle* exercise in healthy volunteers. On the contrary, E_A_ has been shown to increase in an intensity-dependent manner in ET athletes [44] and healthy volunteers [31] *during cycle exercise, and in subjects with hypertension and normal blood pressure during isometric handgrip exercise [45]. The conflicting results can be explained by the dependency of E_A_ on the type and intensity of exercise. Hemodynamically, static (isometric) exercise causes pressure load with accompanied increased E_A_, while dynamic (isotonic) exercise causes volume load with little change in arterial pressure [46]. However, E_A_ may increase during dynamic exercise at high intensities due to increase in cardiac output [47].*

**R2C9:** I disagree with the authors discussion section on VA coupling during exercise. Authors need to be careful about how they define VA coupling; in this manuscript, it is defined as EA/EES. Other authors have reported VA coupling as EES/EA. This is relevant inasmuch as during exercise, EES increases and authors found that EA decreases. This change does not imply, at all, that there is VA uncoupling. Rather, it demonstrates an appropriate augmentation in contractility. However, because in this manuscript, VA coupling is defined as EA/EES, the value of this ratio declines during exercise. however, the decline in this ratio should not lead to the conclusion that the ventricle is becoming uncoupled; if the ratio were defined as EES/EA, then the ratio goes up. Please revise this discussion section appropriately.

**Response:** We agree with the reviewer that clear definitions are of high importance. We acknowledge that ventricular-arterial coupling has been defined as both E_ES_/E_A_ and E_A_/E_ES_ in the literature. In this study, ventricular-arterial coupling was defined as E_A_/E_max_ (note that E_ES_ = E_max_), as in the pioneering work by Sunagawa et al.

We have reviewed the discussion section on ventricular-arterial coupling during exercise and changed the wording so that the ratio E_A_/E_max_ decreases, in order to avoid misinterpretations like VA-uncoupling (page 24, lines 473 – 476).

**Updated text:** The decrease in *E_A_/E_max_* seen during exercise in our study indicates that*,* during exercise*,* the cardiovascular system is approaching optimal ventricular efficiency rather than *maximal SW* (defined as when E_max_ is equal to E_A_).

**R2C10:** Authors should include in the discussion why they chose to study ET for this exploratory analysis. The objective of the study was to demonstrate that PV analysis can be obtained noninvasively. It was not to compare exercise hemodynamics in healthy controls v. elite athletes. Why even include endurance athletes here? from a clinical standpoint, it would have been more informative to generate noninvasive PV loops on patients with unexplained dyspnea, for example, or even heart failure prior to and following a drug intervention. Why were endurance athletes included?

**Response:** We have added a rational to why the study population was chosen to include endurance-trained individuals and sedentary controls (page 5, lines 88 – 93).

**Updated text:** *The rationale of recruiting ET for this feasibility study was that this study population was expected to be comfortable with multiple cycling bouts for a prolonged duration and breath-holds in an exercised state, minimizing the risk of the subject not being able to complete the exercise protocol. The SC were then recruited to study the impact of endurance training on cardiovascular hemodynamics using the proposed method.*

**R2C11:** Throughout the manuscript, and in the abstract, specify that you are referring to the left ventricle (LV), since there has been a great deal of emphasis placed on right ventricular (RV) PV analysis – see Tedford’s data from MUSC/Hopkins, Cornwell’s data from Colorado, Brener’s Circ:HF review, etc.

**Response:** We have clarified in the study aim that the proposed method refers to LV PV loops. We have also changed the variable names throughout the manuscript, in the tables, in the figures, and in the abstract to specify that we are referring to the left ventricle.

**R2C12:** Authors go back and forth between using “ET” for endurance-trained, and spelling out endurance-trained. After the acronym “ET” is introduced the first time, use ET consistently throughout the manuscript.

**Response:** We have updated the whole manuscript so that the acronym ET is consistently used instead of endurance-trained after being introduced the first time. Likewise, we have also updated so that the acronym PV is used throughout the manuscript instead of spelling out pressure-volume.

**R2C13:** The limitations section is nicely described – authors nicely emphasize the limitations of their model, particularly the discussion on limitations of assuming Vo = 0. It should also be noted that this assumption is entirely theoretical and in reality, is only relevant when considering the diastolic limb of the pressure-volume curve. It is impossible for pressure to be 0 during systole, so there is no physiologic basis for a Vo = 0 for systole.

**Response:** We have updated the description of V_0_ in the “Pressure-volume loop parameters” section under Method (page 9, lines 172 – 174) and the discussion on V_0_ under the Limitations section (page 25, lines 493 – 497). V_0_ is the volume axis intercept (i.e. the theoretical LV volume when LV pressure is 0 mmHg), thus V_0_ is neither a point in the cardiac cycle, nor a physiological possible state, but rather a theoretical concept.

**Updated text (1/2):** […] and V_0_ is the *volume axis intercept (i.e. the theoretical LV volume when LV pressure is 0 mmHg).* V_0_ was assumed to be zero mmHg [22].

**Updated text (2/2): […]** the assumption that V0 = 0 *ml* during different hemodynamic conditions has not been validated during exercise, *although V0 has been found to be independent of contractile state [25]*. *It should be noted that this is a theoretical assumption, as the LV volume physiologically can never be 0 ml, and that it is only relevant when considering the diastolic part of the PV loop.*

Reviewer 3: Review of ”Endurance-trained subjects and sedentary controls increase ventricular contractility and efficiency during exercise–feasibility of hemodynamics assessed by non-invasive pressure-volume loops” by Östenson et al.

In the above-mentioned study, the authors assessed endurance-trained athletes and sedentary controls at rest and during exercise with non-invasive pressure-volume loops generated by CMR and NIBP using Segment (a software from Medviso, Sweden).

The aim of the study was to assess if pressure-volume loops could be obtained during exercise.

Twenty-three subjects, 13 endurance-trained athletes, and 10 sedentary controls matched for age and gender were included. They were examined at rest and at moderate exercise intensity.

Pressure volume loops at rest and during exercise were computed using input data from a combination of volumetric measurements obtained from the CMR data and ESP from non-invasive brachial blood pressure. End-diastolic pressure was inputted by the user.

End-systolic elastance (Ees) and Effective arterial elastance (Ea) were found using single-beat technique.

The authors concluded that the method is feasible and a new useful tool to assess hemodynamics even during exercise.

The manuscript is well written. The research group is well known for its CMR capabilities.

Cardiac function and physiology have been studied intensively for decades. Pressure-Volume loops are an accurate method to describe cardiac function. The gold standard for obtaining PV-loops is measuring pressure and volume simultaneously in the ventricle using an admittance/conductance catheter. Combining CMR (the gold standard for volume assessment) with pressure has great potential in research and in the clinic.

Therefore, the method proposed by this group of researchers is of great interest.

I do have some limitations.

**R3C1:** The equations used in the methods section are difficult to follow.

In order to increase impact and readability may I suggest using End-systolic pressure (ESP) instead of LVP max elastance. To use end-systolic volume (ESV) instead of LVV max elastance.

The authors use EDV for end-diastolic volume in equation 6 already.

Like Borlaug and Kass in fig 1.

Figure 1 Heart Fail Clin 2009;5(2):217-28

**Response:** Thank you for the suggestions. We have updated the equations to increase impact and readability (equations 2, 7, 8).

**Updated equations:**

$PE=V(T_{LVP,max})\times\frac{LVESV-V_{0}}{2}$	(2)

$E_{\max}=\frac{\mathrm{LVESP}}{V(T_{LVP,max})-V_{0}}$	(7)

$E_{A}=\frac{\mathrm{LVESP}}{LVEDV-LVESV}$	(8)

**R3C2:** Why don’t the authors rewrite equation 11 to EA/EES = ESV/SV, which is extremely precise using CMR. (and closely related to LVEF).

**Response:** We have added the rewritten equation 11 (equation 12 in the revised manuscript) as equation 13 in the revised manuscript (page 11, line 221).

**Updated equation:**

$\frac{E_{A}}{E_{\max}}\approx\frac{\mathrm{LVESP}/\mathrm{LVSV}}{\mathrm{LVESP}/\mathrm{LVESV}}=\frac{\mathrm{LVESV}}{\mathrm{LVSV}}$	(13)

**R3C3:** I must admit I have problems with the user input for EDP. It is a guess. LVEDP can be above 15 mmHg, especially in decompensated heart failure patients.

**Response:** Thank you for sharing your concern regarding the assumption of LVEDP. As stated in the Limitation section, Seemann et al. have performed a sensitivity analysis of LVEDP ranging from 0 – 15 mmHg resulting in low bias and variability compared to invasive measures. The LVEDP range of 0 – 15 mmHg can be considered physiological. However, as pointed out, LVEDP can exceed 15 mmHg in pathologic disorders. This study demonstrates feasibility of non-invasive PV loops during exercise from CMR in a healthy study population, but not in a study population with LVEDP outside physiological ranges. We have added a clarification about the generalizability of the results (page 25, lines 504 – 506).

**Updated text:** *It should be noted, however, that there are pathological conditions where LVEDP exceeds 15 mmHg, for example decompensated heart failure [51]. The results from this study may not be generalized to a population with pathological LVEDP.*

**R3C4:** Brachial blood pressure was measured non-invasively. However, it is unclear if the pressure was a. derived using pulse-wave analysis, which reflects central blood pressure and hence ESP, or b. from standard brachial sphygmomanometry, which doesn’t reflect central BP/ESP and has for many decades been a poor surrogate for aortic pressure due to pressure amplification (fig 2 McEniery et al). If so, it is a limitation and should be addressed. In the case of the later multiplication of 0.9 has been used to derive ESP.

Figure 2 Pressure amplification Eur Heart J. 2014 Jul 7; 35(26): 1719–1725.

**Response:** Brachial blood pressure was measured non-invasively using an automatic brachial sphygmomanometer (Invivo Expression, Invivo Corporation, Orlando, FL, USA). We acknowledge that brachial blood pressure does not reflect LV pressure, as illustrated in the figure by McEniery et al. However, the proposed method accounts for this fact, as described by Seemann et al. (Circ Cardiovasc Imaging 2019) when developing and validating the method. In their study, the time-varying elastance curve was modeled by the analytical double-Hill equation using data from 875 invasive PV loops. LV pressure throughout the cardiac cycle can be calculated from the time-varying elastance model by inputting 1) brachial pressure and 2) user-determined range of LV end-diastolic pressure. LVESP is approximated from brachial pressure according to the equation proposed by Kelly et al. Circulation 1992):

$$LVESP=\frac{2}{3}SBP+\frac{1}{3}\mathrm{DBP}$$

where SBP is systolic blood pressure and DBP is diastolic blood pressure.

A brief summary of the algorithm above has been added to “Pressure-volume loop parameters” in the Methods section (page 8, lines 147 – 160) for clarification.

**Updated text:** LV PV loops at rest and exercise were computed using a *previously developed and validated* plugin analysis tool in the software Segment [10]. *In brief, the LV time-varying elastance curve was modeled by the analytical double-Hill equation. The parameters in the double-Hill equation were optimized by data from 875 invasively measured elastance curves. The amplitude of the modeled LV elastance curve is defined by left ventricular end-systolic pressure (LVESP) and end-diastolic pressure (LVEDP). LVESP can be approximated by non-invasive brachial sphygmomanometer measures of systolic blood pressure (SBP) and diastolic blood pressure (DBP) [18]:*

$LVESP=\frac{2}{3}SBP+\frac{1}{3}\mathrm{DBP}$	(1)

The plugin requires user-input assumption of LVEDP, which was set to 0 – 15 mmHg according to data from previous studies [19–21]. *LV blood pressure over the cardiac cycle is calculated from the modeled LV elastance curve and time-resolved CMR volumetric measurements, and the final output is the PV loop.*

**R3C5:** For equation 9 (the standard SVR equation), the authors assume CVP is = 0 mmHg. CVP is rarely 0 mmHg, and I think it is an error to do so. The equation should be rewritten to MAP/CO = total peripheral resistance.

**Response:** We have rewritten equation 9 (equation 10 in the revised manuscript) (page 10, line 212).

**Updated text:**

$SVR=\frac{\mathrm{MAP}}{\mathrm{CO}}$	(10)

**R3C6:** The authors refer to reference 9 and 10, where they have demonstrated the agreement of PV-loops generated by invasively measured pressures and CMR volumetric data compared to PV-loops generated conductance catheters in pigs. Has the non-invasive method (used here) been compared to an invasive method in humans? Ea/Ees ratio is very precise in CMR due to the relationship between ESV and SV (see [R3C2]), but we don’t know about the individual components (Ea and Ees). The real goal is to compare multiple non-invasive generated PV-loops during changes in preload or afterload.

**Response:** In a collaboration between University of Oxford and our research group, Per Arvidsson *et al.* are conducting a study comparing the proposed non-invasive method of deriving PV loops to an invasive method in humans. The manuscript is currently under review in Circulation: Heart Failure. The abstract was presented at British Cardiovascular Society Conference and European Society of Cardiology Congress (reference: Arvidsson P *et al.* Invasive validation of pressure-volume loops derived from cardiovascular magnetic resonance imaging and brachial blood pressure in heart failure patients. Eur Heart J. 2022; 43 (Supplement): 229). The results from the abstract showed that the non-invasive method had strong correlations and low biases for the PV loop parameters compared to the invasive method. The conclusion was that the method of obtaining non-invasive PV loops using CMR is ready to be used in a research setting. We have updated the Introduction section (page 3, lines 59 – 66).

We agree that it would indeed be interesting to conduct a study where multiple non-invasive PV loops were compared with invasive PV loops under control of preload and afterload.

**Updated text:** A newly developed and *experimentally* validated non-invasive method to calculate left ventricular (LV) PV loops at rest from cardiac magnetic resonance (CMR) images and brachial pressure has recently been presented [9,10]. This method *has later been validated against invasive measurements in humans [11]* and could potentially be used to obtain PV loops also in the setting of exercise.

The editor asked for the following additional requirements:

1. Please ensure that your manuscript meets PLOS ONE's style requirements, including those for file naming. The PLOS ONE style templates can be found at https://journals.plos.org/plosone/s/file?id=wjVg/PLOSOne_formatting_sample_main_body.pdf and

https://journals.plos.org/plosone/s/file?id=ba62/PLOSOne_formatting_sample_title_authors_affiliations.pdf

**Response:** We have updated the style of the manuscript to meet PLOS ONE’s requirements. Author affiliations have been updated to conform to the formatting requirements. All figure files have been renamed to conform to PLOS ONE’s requirements.

2. Please amend your current ethics statement to address the following concerns:

a) Did participants provide their written or verbal informed consent to participate in this study?

b) If consent was verbal, please explain i) why written consent was not obtained, ii) how you documented participant consent, and iii) whether the ethics committees/IRB approved this consent procedure

**Response:** We have amended the ethics statement and added it to the Methods section (pages 3 – 4, lines 69 – 73).

**Updated text:** This single-center, exploratory, observational, prospective study was approved by the regional ethics committee in Lund (Dnr 741/2004, with complementary Dnr 269/2005) and follow the Declaration of Helsinki. *All participants provided their written consent to participate in the study.*

3. We note that you have stated that you will provide repository information for your data at acceptance. Should your manuscript be accepted for publication, we will hold it until you provide the relevant accession numbers or DOIs necessary to access your data. If you wish to make changes to your Data Availability statement, please describe these changes in your cover letter and we will update your Data Availability statement to reflect the information you provide.

**Response:** We have stated in the Data Availability statement that some restrictions will apply due to ethical concerns. We would like to keep our Data Availability statement:

Full data cannot be shared publicly because of the small sample size and the risk of identifying individual subjects. Data are available from the Cardiac MR Group in Lund, Lund University, Sweden for researchers who meet the criteria for access to confidential data and after additional consent from the research participants.
